# A Polymorphism in the Gene Encoding Heat Shock Factor 1 (*HSF1*) Increases the Risk of Type 2 Diabetes: A Pilot Study Supports a Role for Impaired Protein Folding in Disease Pathogenesis

**DOI:** 10.3390/life12111936

**Published:** 2022-11-20

**Authors:** Elena Klyosova, Iuliia Azarova, Alexey Polonikov

**Affiliations:** 1 Laboratory of Biochemical Genetics and Metabolomics, Research Institute for Genetic and Molecular Epidemiology, Kursk State Medical University, 18 Yamskaya Street, 305041 Kursk, Russia; 2 Department of Biology, Medical Genetics and Ecology, Kursk State Medical University, 3 Karl Marx Street, 305041 Kursk, Russia; 3 Department of Biological Chemistry, Kursk State Medical University, 3 Karl Marx Street, 305041 Kursk, Russia; 4 Laboratory of Statistical Genetics and Bioinformatics, Research Institute for Genetic and Molecular Epidemiology, Kursk State Medical University, 18 Yamskaya Street, 305041 Kursk, Russia

**Keywords:** type 2 diabetes mellitus, genetic susceptibility, molecular chaperons, heat shock factor 1 (HSF1), single nucleotide polymorphism, gene expression, protein folding, unfolded protein response, body mass index, obesity, sex dimorphism

## Abstract

The aim of this pilot study was to investigate whether polymorphisms in the gene encoding heat shock factor 1 (*HSF1*), a transcriptional activator of molecular chaperones, play a role in the development of type 2 diabetes (T2D). A total of 3229 unrelated individuals of Slavic origin, including 1569 T2D patients and 1660 age- and sex-matched healthy controls, were enrolled for the study. Five common single nucleotide polymorphisms (SNPs) of the *HSF1* gene were genotyped using the MassArray-4 system. SNPs rs7838717 (*p* = 0.002) and rs3757971 (*p* = 0.005) showed an association with an increased risk of T2D in females with a body mass index ≥ 25 kg/m^2^. The rs7838717T-rs4279640T-rs3757971C and rs7838717T-rs4279640T-rs3757971T haplotypes were associated with increased and decreased disease risk in overweight or obese females, respectively. The associations were replicated as disease susceptibility genes in large cohorts from the UK Biobank (*p* = 0.008), DIAMANTE (*p* = 2.7 × 10^−13^), and DIAGRAM (*p* = 0.0004) consortiums. The functional annotation of the SNPs revealed that the rs7838717-T and rs3757971C alleles correlated with increased expression of the genes involved in unfolded protein response. The present study showed, for the first time, that genetic variation of *HSF1* is associated with the risk of type 2 diabetes, supporting a role for impaired protein folding in disease pathogenesis.

## 1. Introduction

Diabetes mellitus is one of the largest global health problems of the 21st century [1]. The International Diabetes Federation (IDF) has predicted that the prevalence of diabetes mellitus will increase from 10.5% in 2021 to 12.2% by 2045, affecting 537 million people between the ages of 20 and 79 [1]. Russia places second among all European nations in terms of the prevalence of diabetes, with 90% of patients having type 2 diabetes [1]. Type 2 diabetes (T2D) is a chronic disease characterized by hyperinsulinemia, insulin resistance, and pancreatic β-cell failure, with up to 50% cell loss at diagnosis [2]. T2D is a multifactorial disorder determined by interactions between environmental and genetic factors [3].

Preproinsulin serves as the starting point for insulin production in pancreatic β-cells, and it has been estimated that the cell is capable of producing 6000 preproinsulin molecules per second [4]. Newly synthesized proinsulin is folded by introducing the nascent polypeptide into the endoplasmic reticulum (ER), cleaving the signal peptide, and forming three proinsulin disulfide bonds that are known to be evolutionally conserved across vertebrates [5,6]. Proinsulin folding begins in the ER, where the local environment supports proinsulin folding overall and favors its disulfide bonds formation [7]. This process is tightly regulated by the ER stress-response pathways, whose effects on pancreatic β-cells could be beneficial or potentially harmful depending on the state of cellular proteostasis. These pathways represent a part of the unfolded protein response (UPR), activated as a result of an accumulation of unfolded or misfolded proteins in the endoplasmic reticulum, the mechanism by which cells control protein homeostasis [8].

Excessive biosynthesis of proinsulin in the ER and mutations in the coding sequence of the insulin gene affect the ER folding environment, leading to misfolding of the proinsulin molecule [6]. It is assumed that proinsulin misfolding represents a phenotype closely related to an insufficient insulin synthesis and associated with diabetes risk [9]. This association has been observed in rodent models having the proinsulin-misfolding mutants, in humans with Mutant INS-gene-induced Diabetes of Youth (MIDY), and with mutations in the critical ER-resident proteins [10], as well as in patients with type 2 diabetes [9].

It is well known that any protein including proinsulin must be appropriately folded into its three-dimensional structure in order to possess biological function, and protein folding happens naturally without the need for external energy sources [11]. Molecular chaperones, or heat shock proteins (HSP), are special types of proteins that represent a part of the sturdy machinery used by cells to cope with the issue of protein folding, thereby keeping proteins in their functional condition. Molecular chaperones help newly generated proteins to be folded correctly, prevent them from aggregating, and thus maintain cellular protein homeostasis [12].

Expression of chaperones is controlled by the transcription factor heat shock factor protein 1 (HSF1), which coordinates the cellular response to ER stress through the activation of the heat shock response (HSR), increasing the expression of numerous molecular chaperones [13,14,15]. HSF1 is also a significant transcriptional activator of co-chaperones [16] and ubiquitin [17], as well as a coordinating factor in the production of transcriptional and translational regulators, signaling molecules, and mitogens in response to stress [18,19]. Moreover, HSF1 is assumed to act as a cellular defender against protein deterioration, misfolding, and aggregation in both the cytoplasm and nucleus [20]. Despite the fact that HSF1 activates almost all chaperones and regulates UPR, the genetic variability of this chaperone has never been considered as a potential factor that may contribute to the development of type 2 diabetes. Taking into account the critical role of heat shock factor protein 1 in proteostasis, polymorphisms in the *HSF1* gene represent attractive targets for investigation of their association with the risk of T2D. Therefore, the aim of this pilot study is to investigate whether single nucleotide polymorphisms (SNP) of the *HSF1* gene are associated with the risk of type 2 diabetes.

## 2. Materials and Methods

### 2.1. Study Population

The study was conducted on an ethnically homogeneous population of Central Russia, including unrelated residents who were all of Slavic origin. The study was designed in accordance with the STREGA (STrengthening the REporting of Genetic Association Studies) guidelines. The overall sample size is 3229 individuals, of which 1569 patients with T2D (586 males and 983 females) were on treatment at the Endocrinology Division of the Kursk City Clinical Emergency Hospital from November 2016 to October 2019. T2D patients’ mean age was 61.1 ± 6.9 years. The control group included 1660 healthy donors (631 males and 1029 females) recruited at the Regional Blood Transfusion Station in our previous studies [21,22] with an average age of 60.8 ± 5.7 years. 

Each participant signed their informed consent prior to being included in the study. The regional ethics committee of the Kursk State Medical University approved the study protocol (protocol No. 10, dated 12 December 2016). The diagnosis of type 2 diabetes mellitus was verified by experienced endocrinologists according to the WHO guidelines [23,24]. The validated questionnaire was used to interview all participants for disease-related risk factors [25].

### 2.2. Genetic Analysis

Five milliliters of fasting venous blood were drawn from all study patients into Vacuette vacuum tubes containing 0.5 mM EDTA for genetic analysis. Isolation of genomic DNA was carried out by phenol-chloroform extraction and a column-based method with the QIAamp DNA blood mini kit (QIAGEN, Germany). The purity, quality, and concentration of the isolated DNA solution were assessed using a NanoDrop spectrophotometer (Thermo Fisher Scientific, Waltham, MA, USA). The SNPinfo bioinformatics tools such as GenePipe and FuncPred (https://snpinfo.niehs.nih.gov (accessed on 9 February 2022)) were used for selection of SNPs of the *HSF1* gene. SNPs were selected based on GenePipe’s tool default settings (genotype data from HapMap, the CEU population, and a minor allele frequency cutoff value of 0.10), with a minimum of two SNPs (r^2^ ≥ 0.8) being tagged by each tag SNP (date of access 15 January 2022). The FuncPred tool (date of access 10 February 2022) was used for functional SNP annotation. In total, five common SNPs of the *HSF1* gene, namely rs12542298, rs7838717, rs4279640, rs3757971, and rs7827865 were selected for the genotyping. SNP rs4279640 was selected as a tagSNP. The combination of selected SNPs satisfied the conditions for their iPLEX-based co-genotyping in a single multiplex panel. Genotyping of the polymorphisms was done using the MassARRAY-4 genetic analyzer (Agena Bioscience, San Diego, CA, USA). To assure quality control, 95 randomly selected DNA samples were chosen without knowledge of the case-control status for repeat genotyping on the same platform, and the repeatability test yielded a 100% concordance rate. Since SNPs such as rs12542298 and rs7827865 yielded too low a genotyping call rate (<50%), they were excluded from the statistical analysis. 

### 2.3. Biochemical Analysis

Six mL of fasting venous blood from 426 T2D patients and 136 healthy subjects was drawn for biochemical investigations of glutathione and reactive oxygen/nitrogen species using the Varioscan Flash microplate reader (Thermo Fisher Scientific, USA). Glutathione levels were measured using the OxiSelectTM Total Glutathione (GSSG/GSH) Assay Kit (Cell Biolabs, San Diego, CA, USA). The ROS/RNS levels were measured using the OxiSelectTM In Vitro ROS/RNS Assay Kit (Cell Biolabs, USA). The concentration of glucose, glycated hemoglobin, total cholesterol, high- and low-density lipoproteins, and triglycerides were assessed using the semi-automatic biochemical analyzer Clima MC-15 (RAL, Sevilla, Spain) and reagent kits from Diacon-DS (Moscow, Russia). The Cobas 6000 Roche Diagnostics (Basel, Switzerland) analyzer was used for measuring the plasma concentration of C-peptide by a competitive solid-phase chemiluminescent enzyme immunoassay.

### 2.4. Statistical Methods

The genetic association study power calculator, accessible online at http://csg.sph.umich.edu/abecasis/gaspowercalculator/ (accessed on 11 January 2022), was used to calculate the statistical power for the study. Association analysis between the *HSF1* gene polymorphisms and the risk of T2D could detect the genotype relative risk of 1.24–1.49 assuming 0.85–0.95 power and a 5% type I error (α = 0.05) on the sample size of 1569 cases and 1660 controls. Allele and genotype frequencies in cases and controls were counted and compared by the chi-square test with the values predicted by the assumption of the Hardy–Weinberg equilibrium. Associations between SNPs and T2D risk were evaluated by multiple logistic regression analysis with adjustments for covariates such as sex, age, and body mass index (BMI) using the SNPStats statistical software (https://snpstats.net (accessed on 20 May 2022)). All sex- and BMI-stratified calculations were adjusted for age. Replication analysis of SNP–T2D associations was performed using large scale genomic data from UK Biobank (http://geneatlas.roslin.ed.ac.uk, date of access 21 September 2022) and the T2D Knowledge Portal (https://t2d.hugeamp.org, date of access 21 September 2022). Linkage disequilibrium (LD) measures such as Lewontin’s *D* and *D*’ were calculated with the LDpair Tool (https://ldlink.nci.nih.gov, accessed on 27 September 2022) using genotype data from the 1000 Genomes Project. The quantitative biochemical parameters were initially examined for normality by the Kolmogorov–Smirnov test using the STATISTICA software (v13.3, USA). Since the biochemical parameters showed a deviation from normal distribution, they were expressed as median (Me) and first and third quartiles [Q1; Q3]. Associations between polymorphisms of the *HSF1* gene and rank-based inversely normal transformed biochemical parameters were analyzed by linear regression analysis using software SNPStats; *p* ≤ 0.05 was considered statistically significant.

### 2.5. Functional Annotation of SNPs

The eQTL analysis of the SNPs was performed using genome–transcriptome data from the following databases: (1) the eQTLgen consortium (https://www.eqtlgen.org, date of access 3 October 2022), which includes data from 30,847 blood samples from relatively healthy donors; and (2) the GTEx portal database (https://www.gtexportal.org, date of access 3 October 2022). Tissues of interest that were related to T2D pathogenesis, namely the pancreas, skeletal muscle, and visceral adipose tissue, were selected for the eQTL analysis. The *Enrichr* bioinformatics tools (https://maayanlab.cloud/Enrichr, date of access 12 October 2022) were used to identify biological functions of molecular chaperones of interest using Gene-Ontology-based overrepresentation analysis. 

## 3. Results

### 3.1. Association of HSF1 Gene Polymorphisms with the Risk of Type 2 Diabetes

The baseline, clinical, and laboratory characteristics of the study participants are described previously [26]. Genotype frequencies for all polymorphisms were in Hardy–Weinberg equilibrium in both cases and controls. Table 1 presents the results of association analysis of alleles and genotypes of the *HSF1* gene polymorphisms with susceptibility to type 2 diabetes in both entire and sex-stratified groups. The rs3757971-C/C genotype is associated with an increased risk of T2D (OR = 1.30, 95% CI 1.03–1.64, *p* = 0.026). A sex-stratified analysis showed that a carriage of both the rs3757971-C/C genotype (OR = 1.42, 95% CI 1.04–1.95, *p* = 0.027) and the rs7838717-T/T genotype (OR = 1.53, 95% CI 1.12–2.08, *p* = 0.0078) was associated with disease risk only in females. Moreover, the rs3757971-C and rs7838717-T alleles showed significant associations with T2D susceptibility.

Since obesity is a well-recognized confounding risk factor for T2D [27], it would be reasonable to analyze associations in groups stratified by body mass index. Pursuing this interest, the study patients were subdivided into the two groups. The first included subjects with BMI ≤ 25 kg/m^2^ (i.e., normal body weight), while the second comprised subjects who were overweight or obese (i.e., BMI ≥ 25 kg/m^2^). Following this, a BMI-stratified analysis adjusted for age (Table 2), revealed significant associations between genotypes such as rs7838717-T/T (OR = 1.62, 95% CI 1.20–2.19, *p* = 0.0016) and rs3757971-C/C (OR = 1.54, 95% CI 1.14–2.09, *p* = 0.0047) and an increased risk T2D, but only in overweight and obese females. In males, no statistically significant associations were observed. Linear regression analysis allowed associations to be revealed between polymorphisms of the *HSF1* gene and some biochemical parameters in T2D patients (Supplementary Table S1). A decrease in blood glucose after the first meal (i.e., breakfast) was associated with the rs7838717-T/T genotype (*p* = 0.027) in the entire group analysis. 

The rs3757971-C/C genotype in females was associated with a decreased level of plasma low-density lipoproteins (*p* = 0.033). The rs4279640-C/C genotype was associated with decreased levels of triglycerides in females (*p* = 0.039). In addition, an association of genotype rs4279640-C/C with a decreased glomerular filtration rate was established in both entire (*p* = 0.035) and female (*p* = 0.021) groups. As can be seen from Supplementary Table S1, the above associations were weak in their strength. No statistically significant associations of HSF1 polymorphisms with biochemical parameters were found in males.

### 3.2. HSF1 Haplotypes and T2D Susceptibility

The frequencies of *HSF1* haplotypes in T2D patients and healthy controls are shown in Table 3. Three common haplotypes of *HSF1* with a frequency of more than 13% were identified. As can be seen from Table 3, the rare haplotype TTT (H6) of *HSF1* was associated with decreased risk of T2D in the entire group (OR = 0.51, 95% CI 0.27–0.98, *p* = 0.043). Sex-stratified analysis showed that the common haplotype TTC (*H2*) was associated with increased risk of T2D in females (OR = 1.24, 95% CI 1.04–1.48, *p* = 0.014). A joint BMI- and sex-stratified analysis (Table 4) showed associations of *HSF1* haplotypes with T2D risk in both males and females who were overweight or obese. In particular, the *H2* haplotype was associated with increased risk of type 2 diabetes in females (OR = 1.21, 95% CI 1.02–1.43, *p* = 0.02). In addition, haplotype *H6* showed an association with decreased disease risk in females with BMI more than 25 kg/m^2^ (OR = 0.35, 95% CI 0.15–0.83, *p* = 0.02). In males who were overweight or obese, the CTT haplotype (*H3*) was found to be associated with decreased risk of T2D (OR = 0.74, 95% CI 0.56–0.98, *p* = 0.02).

Table 5 shows the values of linkage disequilibrium between SNPs in the *HSF1* gene. The studied SNPs were in linkage disequilibrium with each other to varying degrees, and strong differences in the LD values were observed between the Russian and European populations of the 1000 Genomes Project. The rs3757971 and rs7838717 polymorphisms are negatively linked to each other in our population, but positively linked in Europeans. A strong inter-population difference was also seen in the *D*-values between SNPs rs3757971 and rs4279640, which were in the negative linkage disequilibrium in our population and positive in the European population. Furthermore, both in our population and in Europeans from the 1000 Genomes Project, the rs7838717 polymorphism was found to be in negative linkage disequilibrium with rs4279640. 

### 3.3. The Replication Analysis for SNP–T2D Associations in Independent Populations

Replication analysis of associations between the studied *HSF1* gene variants and T2D phenotypes was carried out in large populations from the T2D Knowledge portal and the UK Biobank. The results of the replication analysis are presented in Table 6. It is important to note that associations of SNPs rs3757971 and rs7838717 with T2D susceptibility, originally established in our population, were successfully replicated in independent populations. However, associations of these SNPs have not been confirmed as T2D susceptibility markers in a subpopulation of the type 2 diabetics from the UK Biobank. Moreover, the rs4279640 polymorphism showed association with a decreased risk of T2D in some of the studied cohorts, whereas we did not see such an association in our population.

### 3.4. Functional SNP Annotation

The results of the eQTL analysis for the studied *HSF1* gene polymorphisms are shown in Table 7. SNPs rs7838717 and rs3757971 were associated with increased expression of the *VPS28* gene both in the blood (*p* = 1.9 × 10^−57^ and *p* = 1.7 × 10^−66^, respectively) and skeletal muscles (*p* = 2.6 × 10^−5^ and *p* = 1.3 × 10^−4^, respectively). Polymorphisms rs7838717 and rs3757971 were associated with a decrease in the *DGAT1* gene expression (*p* = 8.7 × 10^−33^ and *p* = 1.1 × 10^−35^, respectively). SNP rs7838717 was also associated with decreased levels of the *SHARPIN* gene (*p* = 7.8 × 10^−8^). Increased blood expression of the *MAF1* gene was associated with SNP rs7838717 (*p* = 8.8×10^−6^). In subcutaneous adipose tissue, polymorphisms rs7838717 (*p* = 4.9 × 10^−5^) and rs3757971 (*p* = 9.7 × 10^−8^) were associated with increased expression of the *SCX* gene, whereas polymorphism rs4279640 (*p* = 3.3 × 10^−6^) was negatively correlated with expression levels of the *SCX* gene.

The GTEx-calculator (https://gtexportal.org/home/testyourown, date of access 3 October 2022) was used to assess the effects of the T2D-asociated SNPs on the expression levels of molecular chaperones representing the Hsp70 and Hsp90 families, which are known targets of *HSF1* in T2D-related tissues such as the pancreas, skeletal muscle, and adipose tissue (Table 8). We found that a decrease in expression levels of chaperones such as *HSP90B1*, *RPS19BP1*, and *HSPA5* was associated with a carriage of the rs3757971-C and/or rs7838717-T alleles that were found to be associated with the risk of T2D in our study. Interestingly, these chaperones are directly involved in many biological processes such as ATF6-mediated UPR (GO:0036500), de novo post-translational protein folding (GO:0051084), cellular response to glucose starvation (GO:0042149), cellular response to topologically incorrect protein (GO:0035967), proteasome-mediated ubiquitin-dependent protein catabolic process (GO:0043161), negative regulation of apoptotic process (GO:0043066), post-translational protein modification (GO:0043687), and some others. The disease-associated allele rs7838717-T correlated with increased expression of the *NFE2L2* gene (transcription factor playing a key role in the response to oxidative stress by binding to antioxidant response elements in the promoters of many cytoprotective genes), which is involved in the activation of UPR and responsible for multiple biological functions, such as cytokine stimulus (GO:0071345), hydrogen peroxide (GO:0070301), oxidative stress (GO:0034599), ER-associated ubiquitin-dependent protein catabolic process (GO:0000058), positive regulation of the ERAD pathway (GO:1904294), and response to tumor necrosis factor (GO:0034612). 

## 4. Discussion

The present study found, for the first time, that polymorphisms of gene encoding heat shock factor 1 are associated with an increased risk of type 2 diabetes. However, sex and body mass index were found to be confounding factors, modifying the associations between the polymorphisms and T2D risk. In particular, SNPs such as rs7838717 and rs3757971 were found to be associated with an increased risk of T2D in females with a BMI ≥ 25 kg/m^2^. The observed associations were successfully replicated as disease-susceptibility markers in large cohorts from the UK Biobank, DIAMANTE, and DIAGRAM consortiums. In the MAGIC and TOPMed consortiums, these SNPs were also found to be associated with increased fasting blood glucose (FBG) adjusted by BMI, although we did not see an association of the SNPs with FBG in our population. Two haplotypes, such as rs7838717T-rs4279640T-rs3757971C and rs7838717T-rs4279640T-rs3757971T of *HSF1*, showed associations with increased and decreased risk of type 2 diabetes in overweight or obese females, respectively. In contrast, the rs7838717C-rs4279640T-rs3757971T haplotype was associated with decreased disease risk in males with a BMI ≥ 25 kg/m^2^. The functional annotation of T2D-associated polymorphisms showed that the T2D-associated alleles such as rs7838717-T and rs3757971C were correlated with increased expression of the *CPSF1* gene in the pancreas, skeletal muscle, subcutaneous adipose tissue, and whole blood. In addition, these SNPs were associated with increased expression of *VPS28* in skeletal muscle and blood, as well as with expression levels of some other genes in the blood. 

Many studies have shown that mutations in genes encoding chaperones and co-chaperones may cause different diseases, such as neuromuscular diseases [28], neurodegenerative disease [29], and Alzheimer’s disease [30]. Studies investigating the association between *HSF1* gene polymorphisms and T2D susceptibility have not been done so far. Nonetheless, several studies have been undertaken to assess the relationship between polymorphisms of molecular chaperones and the development of type 2 diabetes. In particular, Synofzik et al. have shown that loss-of-function mutations of the *DNAJC3* (DnaJ heat shock protein family (Hsp40) member C3) gene contribute to the development of diabetes mellitus in humans [29]. In a study by Moniruzzaman M et al., it was found that the +2437T/C polymorphism (rs2227956) of *HSPA1L* is significantly associated with the incidence of type 2 diabetes in the population of Bangladesh [31]. Elshahed O.M. et al. observed significant differences in the prevalence of haplotypes such as CGGT, CCGT, AGGT, and AGAT of the *HSPA1A* gene between diabetic patients with nephropathy and healthy controls [32].

It is known that heat shock factor 1 is a transcription factor that promotes UPR and binds to heat shock elements (HSEs) in the promoter regions of HSPs, which are necessary for directing damaged and misfolded proteins toward proteasomal degradation [33]. Certain data in the literature indicate changes in the expression levels of HSF1 in type 2 diabetes mellitus. Kavanagh et al. [34] experimentally show that pancreatic cells of monkeys with T2D have increased expression of *Hsf1*. This finding was confirmed by the study of Marselli L et al. [35], who investigated the expression profile of pancreatic β-cells from 9 patients with T2D and 10 non-diabetic controls. The authors revealed that expression of *HSF1* in pancreatic β-cells was significantly increased in patients with T2D compared to controls. We hypothesize that the increased expression of the *HSF1* gene may mirror the need of the β-cells in the synthesis of more molecules of heat shock factor 1. It is known that *HSF1* is required to ensure normal protein folding through the activation of the molecular chaperone cascade, including the Hsp70 and Hsp90 families [20,36]. This assumption is in line with studies highlighting the importance of impaired protein folding for the development of type 2 diabetes [37,38,39]. The pancreas is an organ with increased rates of protein synthesis, and therefore higher chaperone levels are required to ensure proper folding of proteins, including proinsulin. Notably, the decreased transcriptional activity of *HSF1* was found to enhance glucolipotoxicity-induced apoptosis in both rat and human β-cells [40], suggesting a role of heat shock factor 1 in the initial mechanisms underlying type 2 diabetes mellitus.

As can be seen from Table 5, SNPs rs7838717 and rs3757971 are in positive LD with each other, whereas these SNPs are correlated negatively with rs4279640, a polymorphism which did not show association with T2D. The T2D-associated variant alleles (rs7838717-T and rs3757971-C) are correlated with the wild-type rs4279640-T allele. Apparently, the association of rs7838717 and rs3757971 with T2D risk can be explained by the relationship of these polymorphisms with the expression levels of genes such as *VPS28*, *KIAA1875*, *TONSL*, *EPPK1*, and *BOP1* (these genes were not correlated with SNP rs4279640). However, this assumption should be investigated in experimental studies.

Although the disease-associated alleles were not correlated with changes in *HSF1* gene expression, they showed correlations with the expression of genes involved in the regulation of proteostasis and unfolded protein response. In particular, it is known that *VPS28* is a component of the ubiquitin–proteasome pathway and is important for lysosomal targeting [41]. *VPS28* is also involved in protein transport into vesicles and ubiquitin-dependent catabolism through the sorting of multivesicular bodies [42]. Thus, *VPS28* eliminates dysfunctional/misfolded proteins through the ubiquitin–proteasome pathway, thereby regulating cellular proteostasis. Thus, we suggest that an increase in the expression of the *VPS28* gene in subjects with the T2D-associated alleles may demonstrate the increased activation of the ubiquitin–proteasome pathway, a part of the unfolded protein response directed at the degradation of unfolded or misfolded proteins in the ER, the conditions playing a role in the pathogenesis of type 2 diabetes [43,44,45].

The rs7838717-T and rs3757971-C alleles were also correlated with a decreased expression of *DGAT1*, which encodes the enzyme diacylglycerol O-acyltransferase 1, catalyzing the synthesis of triacylglycerol from diacylglycerol (DAG) and acyl-CoA as substrates [46]. Interestingly, DAG is known to be a lipid signal molecule playing a physiological role in β-cells; in particular, in the regulation of insulin secretion [47] and also participating in the induction of apoptosis [48].

The *SHARPIN* gene was also of interest, whose decreased expression was associated with the carriage of allele rs7838717-T. This gene is a component of the LUBAC complex, which conjugates linear polyubiquitin chains in a head-to-tail manner to substrates and plays a key role in NF–kappa B activation and regulation of inflammation [49,50,51]. The NF–kappa B (NF–κB) pathway is known to play a crucial role in the pathogenesis of T2D and its complications. This transcription factor is activated by a number of pro-inflammatory cytokines to regulate β-cell survival and death in T2D [52]. The LUBAC complex regulates canonical Wnt signaling [53], a pathway linked to insulin resistance, inflammatory response regulation, and dysfunction of pancreatic β-cells and endothelial cells [54]. 

The expression of the *MAF1* gene in blood was correlated with the T2D-associated alleles of *HSF1*. It is known that *MAF1* is a global repressor of RNA polymerase III transcription that regulates the expression of highly abundant noncoding RNAs in response to nutrient availability and cellular stress [55]. Bonhoure N. et al. showed that the knockout of *Maf1* in mice conferred resistance to diet-induced obesity and nonalcoholic fatty liver disease by reducing food intake and increasing metabolic inefficiency [55]. These findings suggest that the increased levels of the *MAF1* gene may be linked to the changes in lipid metabolism occurring in diabetes mellitus and obesity.

Finally, it was a very interesting finding that the rs3757971-C and rs7838717-T alleles of *HSF1* were also correlated with decreased expression of molecular chaperones such as *HSP90B1, HSPA5,* and *FKBP4*—members of the Hsp70 and Hsp90 families and the primary targets for heat shock factor 1 [56]. This finding may suggest that the carriage of T2D-associated alleles of *HSF1* may lead to the decreased expression of these chaperones responsible for efficient folding of proteins, including proinsulin in the pancreas. However, experimental studies are required to reproduce these molecular consequences of *HSF1* deficiency and to draw definitive conclusions about the causal relationship between the *HSF1* gene, molecular chaperones *HSP90B1, HSPA5,* and *FKBP4,* and impaired proinsulin folding in type 2 diabetes.

There are some limitations in the study that should be addressed. We examined a limited number of polymorphisms in the *HSF1* gene, which do not cover all functionally significant sequence variants that may influence expression or activity of this gene. Therefore, further studies with a larger number of SNPs are required to assess the comprehensive contribution of the gene to the risk of T2D. The limited number of male subjects in the sub-group analysis did not allow us to reproduce SNP–disease associations in males. Because the observed associations were relatively weak, more research is needed to assess the relationship between *HSF1* gene polymorphisms and susceptibility to type 2 diabetes in other populations around the world. 

In conclusion, the present pilot study found, for the first time, that genetic variations of heat shock transcription factor 1 contribute to type 2 diabetes susceptibility in females with body mass index ≥ 25 kg/m^2^. The observed associations of polymorphisms rs7838717 and rs3757971 with T2D risk have been successfully replicated in three independent European populations from the UK Biobank, DIAMANTE, and DIAGRAM consortiums. Moreover, two haplotypes such as rs7838717T-rs4279640T-rs3757971C and rs7838717T-rs4279640T-rs3757971T of *HSF1* showed significant associations with T2D risk in overweight or obese females, whereas the rs7838717C-rs4279640T-rs3757971T haplotype was associated with disease risk in males with a BMI ≥ 25 kg/m^2^. A comprehensive bioinformatics analysis showed that the T2D-associated polymorphisms of the *HSF1* genes are linked with the changes in expression of genes involved in the unfolded protein response, a hallmark of the pathogenesis of type 2 diabetes mellitus. The present study provided additional evidence for the role of heat shock transcription factor 1 in the pathogenesis of type 2 diabetes mellitus, and its impact on the disease’s development can be attributed to the impaired folding of proteins, including proinsulin, ultimately leading to the activation of the unfolded protein response. Our study shows that chaperone gene polymorphisms appear to contribute to the development of T2D through disturbances in protein folding and activation of the unfolded protein response, a condition responsible for β-cell loss due to apoptosis [16]. This suggests that heat shock factor 1 could be a promising target for the treatment of type 2 diabetes by improving protein folding and decreasing ER overload from unfolded and misfolded proteins. Further research is warranted to substantiate the molecular mechanisms by which *HSF1* gene polymorphisms are linked to the pathogenesis of type 2 diabetes.

## Figures and Tables

**Table 1:** Genotype and allele frequencies of the HSF1 gene in T2D patients and controls.

SNP	Genotype/ Allele	Healthy Controls n (%) ^1^	Patients with T2D n (%) ^1^	OR ^2^ (95% CI)	*p*-Value ^3^
Entire group					
rs7838717 C>T	C/C-C/T	1467 (88.4)	1345 (85.7)	1.00	0.05
	T/T	193 (11.6)	224 (14.3)	1.26 (1.00–1.59)	
	T	0.36	0.38	1.09 (0.98–1.20)	0.10
rs4279640 T>C	T/T-T/C	1260 (75.9)	1199 (76.4)	1.00	0.65
	C/C	400 (24.1)	370 (23.6)	1.04 (0.87–1.25)	
	C	0.49	0.48	0.97 (0.88–1.07)	0.61
rs3757971 T>C	T/T-C/T	1467 (88.5)	1337 (85.3)	1.00	**0.026**
	C/C	190 (11.5)	231 (14.7)	**1.30 (1.03–1.64)**	
	C	0.35	0.37	1.11 (1.00–1.23)	0.05
Males					
rs7838717 C>T	C/C-C/T	542 (87.8)	513 (87.5)	1.00	0.68
	T/T	75 (12.2)	73 (12.5)	1.08 (0.75–1.56)	
	T	0.38	0.36	0.94 (0.80–1.11)	0.49
rs4279640 T>C	T/T-T/C	485 (78.6)	437 (74.6)	1.00	0.05
	C/C	132 (21.4)	149 (25.4)	1.34 (1.00–1.78)	
	C	0.47	0.50	1.11 (0.95–1.30)	0.20
rs3757971 T>C	T/T-C/T	541 (87.8)	505 (86.2)	1.00	0.42
	C/C	75 (12.2)	81 (13.8)	1.16 (0.81–1.67)	
	C	0.37	0.36	0.97 (0.82–1.15)	0.74
Females					
rs7838717 C>T	C/C-C/T	911 (88.5)	832 (84.6)	1.00	**0.0078**
	T/T	118 (11.5)	151 (15.4)	**1.53 (1.12–2.08)**	
	T	0.35	0.39	**0.17 (1.03–1.33)**	**0.016**
rs4279640 T>C	T/T-T/C	767 (74.5)	762 (77.5)	1.00	0.28
	C/C	262 (25.5)	221 (22.5)	0.87 (0.68–1.12)	
	C	0.49	0.47	0.91 (0.80–1.03)	0.13
rs3757971 T>C	T/T-C/T	912 (88.8)	832 (84.7)	1.00	**0.027**
	C/C	115 (11.2)	150 (15.3)	**1.42 (1.04–1.95)**	
	C	0.34	0.38	**1.19 (1.05–1.36)**	**0.008**

^1^ Absolute number and percentage of individuals/chromosomes with a particular genotype/allele. ^2^ Odds ratio with 95% confidence intervals (crude analysis) with one degree of freedom. ^3^ *p*-Value—significance level. Bold indicates statistically significant *p*-values.

**Table 2:** Genotype and allele frequencies of the *HSF1* gene in T2D patients and controls stratified by sex and BMI.

SNP.	Genotype/ Allele	Healthy Controls n (%) ^1^	Patients with T2D n (%) ^1^	OR ^2^ (95% CI)	*p*-Value ^3^	Healthy Controls n (%) ^1^	Patients with T2D n (%) ^1^	OR ^2^ (95% CI)	*p*-Value ^3^
		Males				Females			
		BMI ≤ 25 kg/m^2^ norm							
rs7838717 C>T	C/C-C/T	104 (90.4)	102 (87.2)	1	0.46	167 (86.1)	73 (83.9)	1	0.96
	T/T	11 (9.6)	15 (12.8)	1.37 (0.60–3.12)		27 (13.9)	14 (16.1)	1.02 (0.48–2.14)	
	T	0.35	0.34	0.99 (0.68–1.45)	0.97	0.35	0.42	1.35 (0.94–1.95)	0.10
rs4279640 T>C	T/T-T/C	90 (78.3)	86 (73.5)	1	0.46	138 (71.1)	70 (80.5)	1	0.14
	C/C	25 (21.7)	31 (26.5)	1.26 (0.69–2.31)		56 (28.9)	17 (19.5)	0.62 (0.33–1.17)	
	C	0.49	0.47	0.95 (0.66–1.36)	0.77	0.50	0.42	0.72 (0.51–1.04)	0.08
rs3757971 T>C	T/T-C/T	101 (87.8)	100 (85.5)	1	0.62	168 (86.6)	71 (81.6)	1	0.67
	C/C	14 (12.2)	17 (14.5)	1.21 (0.57–2.59)		26 (13.4)	16 (18.4)	1.17 (0.57–2.42)	
	C	0.38	0.33	0.82 (0.56–1.20)	0.31	0.33	0.43	1.50 (1.04–2.17)	**0.029**
BMI ≥ 25 kg/m^2^ overweight and obesity									
rs7838717 C>T	C/C-C/T	425 (87.3)	411 (87.6)	1	0.95	735 (89.2)	759 (84.7)	1	**0.0016**
	T/T	62 (12.7)	58 (12.4)	1.01 (0.69–1.49)		89 (10.8)	137 (15.3)	**1.62 (1.20–2.19)**	
	T	0.38	0.37	0.93 (0.78–1.12)	0.46	0.35	0.38	1.15 (1.00–1.32)	0.05
rs4279640 T>C	T/T-T/C	386 (79.3)	351 (74.8)	1	0.11	621 (75.4)	692 (77.2)	1	0.38
	C/C	101 (20.7)	118 (25.2)	1.29 (0.95–1.75)		203 (24.6)	204 (22.8)	0.90 (0.71–1.14)	
	C	0.47	0.50	1.14 (0.95–1.37)	0.15	0.49	0.477	0.94 (0.82–1.08)	0.38
rs3757971 T>C	T/T-C/T	426 (87.7)	405 (86.3)	1	0.54	734 (89.3)	761 (85)	1	**0.0047**
	C/C	60 (12.3)	64 (13.7)	1.13 (0.77–1.65)		88 (10.7)	134 (15)	**1.54 (1.14–2.09)**	
	C	0.37	0.73	1.00 (0.83–1.21)	0.97	0.34	0.37	**1.16 (1.01–1.33)**	**0.038**

^1^ Absolute number and percentage of individuals/chromosomes with a particular genotype/allele. ^2^ Odds ratio with 95% confidence intervals (crude analysis) with one degree of freedom. ^3^ *p*-Value—significance level. Bold indicates statistically significant *p*-values.

**Table 3:** Haplotype frequencies of the *HSF1* gene and their associations with type 2 diabetes.

N	rs7838717	rs4279640	rs3757971	Healthy Controls	Patients with T2D	OR ^1^ (95 CI)	*p*-Value ^2^
**Entire group**							
H1	C	C	T	0.4506	0.4472	1	---
H2	T	T	C	0.3065	0.339	1.07 (0.94–1.21)	0.33
H3	C	T	T	0.1571	0.1485	0.92 (0.78–1.08)	0.31
H4	C	T	C	0.0284	0.0232	0.87 (0.60–1.25)	0.44
H5	T	C	T	0.0246	0.0252	1.00 (0.67–1.49)	1
H6	T	T	T	0.0192	0.0069	**0.51 (0.27–0.98)**	**0.043**
Global p-Value haplotype association: 0.082							
**Males**							
H1	C	C	T	0.43	0.4682	1	---
H2	T	T	C	0.3279	0.3301	0.89 (0.72–1.08)	0.24
H3	C	T	T	0.1585	0.1394	0.81 (0.62–1.05)	0.11
H4	C	T	C	0.0281	0.0231	0.82 (0.46–1.44)	0.49
H5	T	C	T	0.0274	0.019	0.65 (0.34–1.25)	0.19
H6	T	T	T	0.0122	0.0083	0.84 (0.31–2.26)	0.73
Global p-Value haplotype association: 0.44							
**Females**							
H1	C	C	T	0.4607	0.4348	1	---
H2	T	T	C	0.296	0.3442	**1.24 (1.04–1.48)**	**0.014**
H3	C	T	T	0.1567	0.1539	1.00 (0.80–1.25)	0.99
H4	C	T	C	0.0279	0.0232	0.80 (0.49–1.30)	0.37
H5	T	C	T	0.0235	0.0288	1.32 (0.78–2.22)	0.3
H6	T	T	T	0.0233	0.0061	0.43 (0.18–1.05)	0.064
Global *p-*Value haplotype association: **0.027**							

^1^ Odds ratio with 95% confidence intervals (crude analysis) with one degree of freedom. ^2^ *p*-Value—significance level. Gray shading shows minor alleles of SNPs. Bold indicates statistically significant *p*-values.

**Table 4:** Haplotype frequencies of the *HSF1* gene in T2D patients and controls stratified by sex and BMI.

N	rs7838717	rs4279640	rs3757971	Healthy Controls n (%) ^1^	Patients with T2D n (%) ^1^	OR ^2^ (95% CI)	*p*-Value ^3^	Healthy Controls n (%) ^1^	Patients with T2D n (%) ^1^	OR ^2^ (95% CI)	*p*-Value ^3^
				Males				Females			
BMI ≤ 25 kg/m^2^ norm											
H1	C	C	T	0.4482	0.4670	1	---	0.4726	0.4184	1	---
H2	T	T	C	0.3090	0.3068	0.96 (0.63–1.48)	0.87	0.2878	0.4017	1.42 (0.94–2.15)	0.09
H3	C	T	T	0.1456	0.1603	1.07 (0.62–1.83)	0.80	0.1414	0.1327	0.98 (0.54–1.78)	0.95
H4	C	T	C	0.0435	0.0218	0.44 (0.14–1.37)	0.16	0.0350	0.0293	0.62 (0.21–1.85)	0.39
H5	T	C	T	0.0000	0.0155	0.69 (0.17–2.75)	0.60	0.0204	0.0069	0.27 (0.03–2.26)	0.23
H6	T	T	T	0.0279	0.0239	0.95 (0.30–3.07)	0.94	0.0306	0.0110	0.32 (0.06–1.70)	0.18
Global *p-*Value haplotype association: 0.77								Global *p-*Value haplotype association: 0.09			
BMI ≥ 25 kg/m^2^ overweight and obesity											
H1	C	C	T	0.4241	0.4678	1	---	0.4566	0.4363	1	---
H2	T	T	C	0.3332	0.3360	0.91 (0.74–1.12)	0.39	0.2997	0.3386	**1.21 (1.02–1.43)**	**0.02**
H3	C	T	T	0.1618	0.1349	**0.74 (0.56–0.98)**	**0.036**	0.1606	0.1561	1.02 (0.83–1.26)	0.83
H4	C	T	C	0.0255	0.0234	0.85 (0.45–1.59)	0.61	0.0258	0.0226	0.91 (0.56–1.47)	0.70
H5	T	C	T	0.0312	0.0205	0.54 (0.28–1.04)	0.06	0.0242	0.0310	1.40 (0.85–2.31)	0.18
H6	T	T	T	0.0103	0.0036	0.69 (0.33–1.44)	0.32	0.0218	0.0056	**0.35 (0.15–0.83)**	**0.02**
Global *p-*Value haplotype association: 0.13								Global *p-*Value haplotype association: 0.03			

^1^ Absolute number and percentage of individuals/chromosomes with a particular genotype/allele. ^2^ Odds ratio with 95% confidence intervals (crude analysis) with one degree of freedom. ^3^ *p*-Value—significance level. Gray shading shows minor alleles. Bold indicates statistically significant *p*-values.

**Table 5:** Linkage disequilibrium measures between SNPs of the *HSF1* gene in the Russian population and populations of the 1000 Genomes Project.

SNP ID	rs4279640	rs3757971
the Russian population		
rs7838717	−0.1474	0.1972
	0.8253	0.8662
rs4279640	-	−0.1630
	-	0.9321
European populations of 1000 G		
rs7838717	−0.0739	−0.0213
	0.8586	0.8784
rs4279640	-	−0.1630
	-	0.9417

Matrices show LD measures, such as a nonstandardized *D* (upper part) and a standardized *D’* (lower part). LD-values were calculated with the LDpair Tool (https://ldlink.nci.nih.gov (accessed on 27 September 2022)) using genotype data from the 1000 Genomes Project (1000 G) and GRCh37 human genome assembly. Each pair of SNPs includes two LD values calculated for the following populations: the Russian population (upper cells); the European populations of 1000 G (middle cells). All LD values *p* < 0.0001.

**Table 6:** Replication for SNP associations with different T2D phenotypes in large independent cohorts.

			rs7838717 ^1^ C>T		rs4279640 T>C		rs3757971 T>C	
	Phenotype		*p*-Value ^2^	Beta/Odds Ratio	*p*-Value	Beta/Odds Ratio	*p*-Value	Beta/Odds Ratio
UK Biobank ^1^	Non-insulin dependent diabetes n (cases/controls) 19,860/432,404		**0.008**	▼ 0.973	**0.01**	▼ 0.977	**3.5 × 10^−5^**	▲ 1.04
	Type 2 diabetes n (cases/controls) 2889/449,375		0.43	▼ 0.979	0.41	▼ 0.979	0.08	▲ 1.05
T2D Knowledge Portal ^2^	Type 2 diabetes adj BMI	DIAMANTE (European) T2D GWAS n = 157,384	**2.70 × 10^−13^**	▼ 0.944	**5.1 × 10^−6^**	▼ 0.966	**8.2 × 10^−16^**	▲ 1.065
		DIAGRAM 1000G GWAS n = 54,365	**0.00039**	▼ 0.941	**8.6 × 10^−5^**	▼ 0.941	**1.10 × 10^−6^**	▲ 1.083
	Fasting glucose adj BMI	MAGIC 2021 glycemic traits GWAS: Europeans n = 160,378	**0.00004**	▼ −0.009	**2.7 × 10^−4^**	▼ −0.007	**4.9 × 10^−7^**	▲ 0.011
		TOPMed fasting glucose whole genome sequence analysis n =26,807	**0.004**	▼ −0.014	0.17	▼ −0.006	**0.03**	▲ 0.009

^1^ Values are for allele C rs7838717. ^2^ *p*-Value—significance level; values that reached the genome-wide significance level are bolded. ▲ depicts an increased value, ▼ depicts a decreased value. Genomic data obtained at the CVD Knowledge Portal (https://t2d.hugeamp.org), date of access 21 September 2022.

**Table 7:** Relationship between studied SNPs and expression levels of genes in T2D-related tissues.

SNP	Allele	eQTL (Blood)			GTEx-Portal								
		Gene	Z-Score	*p*-Value	Pancreas			Skeletal Muscle			Adipose—Subcutaneous		
					Gene	NES	*p*-Value	Gene	NES	*p*-Value	Gene	NES	*p*-Value
rs7838717	T	*VPS28*	15.97	1.9 ×10^−57^	*CPSF1*	0.33	10^−6^	*CPSF1*	0.15	**1.8 × 10^−4^**	*CPSF1*	0.26	**2.1 × 10^−9^**
	T	*DGAT1*	−11.92	8.7 × 10^−33^				*VPS28*	0.13	**2.6 × 10^−5^**	*SCX*	0.23	**4.9 × 10^−5^**
	T	*KIAA1875*	11.75	7.2 × 10^−32^									
	T	*CPSF1*	10.29	8.1 × 10^−25^									
	T	*TONSL*	7.56	4.1 × 10^−14^									
	T	*EPPK1*	7.27	3.5 × 10^−13^									
	T	*BOP1*	6.69	2.2 × 10^−11^									
	T	*SHARPIN*	−5.37	7.8 × 10^−8^									
	T	*MAF1*	4.44	8.8 × 10^−6^									
rs4279640	C	*DGAT1*	0.10	2.1 × 10^−6^	*CPSF1*	−0.32	3.5 × 10^−6^	*CPSF1*	−0.16	**1.7 × 10^−6^**	*CPSF1*	−0.22	**2.1 × 10^−5^**
	C	*HSF1*	−0.06	2.7 × 10^−6^				*SCRT1*	0.15	**4.5 × 10^−5^**	*SCX*	−0.18	**3.3 × 10^−6^**
	C	*CPSF1*	−0.13	3.8 × 10^−6^									
rs3757971	C	*VPS28*	17.23	1.7 × 10^−66^	*CPSF1*	0.39	1.4 × 10^−7^	*CPSF1*	0.16	**1.3 × 10^−4^**	*CPSF1*	0.29	**2.9 × 10^−7^**
	C	*DGAT1*	−12.46	1.1 × 10^−35^				*VPS28*	0.13	**1.3 × 10^−4^**	*SCX*	0.22	**9.7 × 10^−8^**
	C	*CPSF1*	11.28	1.5 × 10^−29^									
	C	*TONSL*	8.23	1.9 × 10^−16^									
	C	*KIAA1875*	7.09	1.3 × 10^−12^									
	C	*EPPK1*	6.94	3.9 × 10^−12^									
	C	*BOP1*	6.36	2.1 × 10^−10^									
	C	*PPP1R16A*	4.90	9.6 × 10^−7^									

**Table 8:** Relationship between T2D-associated SNPs and expression levels of HSF1-targeted chaperons and their biological functions.

SNP ^1^	Gene ^2^	*p*-Value ^3^	NES ^4^	Gene Ontologies ^5^
rs7838717-T	*NFE2L2*	**0.020**	0.071 ▲	GO:0062197 response to chemical stress; GO:0071345 response to cytokine stimulus; GO:0070301response to hydrogen peroxide; GO:0034599 response to oxidative stress; GO:0071356 response to tumor necrosis factor; GO:0140467 integrated stress response signaling; GO:0036499 PERK-mediated UPR; GO:1903071 positive regulation of ER-associated ubiquitin-dependent protein catabolic process; GO:1904294 positive regulation of ERAD pathway; GO:0010498 proteasomal protein catabolic process; GO:0032446 protein modification by small protein conjugation; GO:1903205 regulation of hydrogen peroxide-induced cell death; GO:1902175 regulation of oxidative stress-induced intrinsic apoptotic signaling pathway; GO:0034612 response to tumor necrosis factor;
rs3757971-C		**0.018**	0.072 ▲	
rs7838717-T	*FKBP4*	**0.0098**	−0.11 ▼	GO:0031345 regulation of cell projection organization; GO:0051494 regulation of cytoskeleton organization; GO:0031111regulation of microtubule polymerization or depolymerization; GO:0018208 peptidyl-proline modification; GO:0000413 protein peptidyl-prolyl isomerization; GO:1900034 regulation of cellular response to heat GO:0080135 regulation of cellular response to stress;
rs7838717-T	*HSP90B1*	**0.0044**	−0.11 ▼	GO:0036500 ATF6-mediated UPR; GO:0044267 protein metabolic process; GO:0006464 protein modification process; GO:0071318 response to ATP; GO:0071345 response to cytokine stimulus; GO:1901701 response to oxygen-containing compound; GO:0019221 cytokine-mediated signaling pathway; GO:1903513 ER to cytosol transport; GO:0036503 ERAD pathway; GO:0043066 regulation of apoptotic process; GO:0043687 post-translational protein modification; GO:0043161 proteasome-mediated ubiquitin-dependent protein catabolic process; GO:0032527 protein exit from ER; GO:0015031 protein transport; GO:0006898 receptor-mediated endocytosis; GO:0042981 regulation of apoptotic process; GO:0010921 regulation of phosphatase activity; GO:0043666 regulation of phosphoprotein phosphatase activity; GO:0034976 response to ER stress; GO:0030970 ER to cytosol; GO:0030433 ubiquitin-dependent ERAD pathway;
rs3757971-C		**0.0012**	−0.12 ▼	
rs3757971-C	*HSPA5*	**0.036**	−0.073 ▼	GO:0051084 de novo post-translational protein folding; GO:0036500 ATF6-mediated unfolded protein response; GO:0042149 cellular response to glucose starvation; GO:0035967 response to topologically incorrect protein; GO:0034620 cellular response to unfolded protein; GO:0051085 chaperone-cofactor-dependent protein refolding; GO:0036503 ERAD pathway; GO:0140467 integrated stress response signaling; GO:0065002 intracellular protein transmembrane transport; GO:0036498 IRE1-mediated UPR; GO:0035437 maintenance of protein localization in ER; GO:0072595 maintenance of protein localization in organelle; GO:0043066 negative regulation of apoptotic process; GO:0051129 regulation of cellular component organization; GO:1900102 regulation of endoplasmic reticulum UPR; GO:0043069 negative regulation of programmed cell death; GO:0031333 negative regulation of protein-containing complex assembly; GO:0036499 PERK-mediated UPR; GO:0030335 positive regulation of cell migration; GO:0006620 post-translational protein targeting to endoplasmic reticulum membrane; GO:0043161 proteasome-mediated ubiquitin-dependent protein catabolic process; GO:0070972 protein localization to ER; GO:0042981 regulation of apoptotic process; GO:0043254 regulation of protein-containing complex assembly; GO:0034976 response to ER stress; GO:0006986 response to unfolded protein;

^1^ SNPs associated with the risk of developing DM2 according to the results of this study; ^2^ Genes involved in metabolic pathways «Protein folding» and «Chaperone» according to databases Reactome (https://reactome.org (accessed on 2 June 2022)), Kegg (https://www.genome.jp/kegg (accessed on 5 June 2022)), and Wikipathways (https://www.wikipathways.org) date of access 8 June 2022; ^3^ *p*-Value—significance level; ^4^ Normalized effect size according to GTEx-calculator (https://www.gtexportal.org), date of access 3 October 2022; ^5^ Gene Ontologies’ biological process according to Enrichr dataset (https://maayanlab.cloud/Enrichr/), date of access 12 October 2022. Bold indicates statistically significant p-values.

## Data Availability

Data supporting reported results are available upon request.

## Supplementary Materials

The following supporting information can be downloaded at: https://www.mdpi.com/article/10.3390/life12111936/s1, Table S1: Association of the studied polymorphic gene Variants with clinical and laboratory parameters in T2D patients.
