# Supplementary material for: A Polymorphism in the Gene Encoding Heat Shock Factor 1 (HSF1) Increases the Risk of Type 2 Diabetes: A Pilot Study Supports a Role for Impaired Protein Folding in Disease Pathogenesis
Source: Life (Basel). 2022 Nov 20;12(11):1936. doi: 10.3390/life12111936 (PMC9694443; doi:10.3390/life12111936)
Supplement: Supplementary file 1 [file life-12-01936-s001.zip › life-2027331-supplementary.pdf]

Supplementary Table S1. Association of the studied polymorphic gene Variants with clinical and laboratory parameters in T2D patients

SNP	Genotype	Entire Group		Males		Females	
		T2D Patients (*n* = 489) Me (Q1; Q3) [1]	*P* [2] *(Q)*	T2D Patients (*n* = 145) Me (Q1; Q3) [1]	*P* [2] *(Q)*	T2D Patients (*n* = 344) Me (Q1; Q3) [1]	*P* [2] *(Q)*
HbA1c							
rs7838717 C>T	C/C	9.00 (7.80; 10.5)	0.21	9.20 (7.8;10.6)	0.40	9.00 (7.8; 10.5)	0.31
	C/T	9.10 (7.7; 11.0)		9.50 (7.9; 11.1)		9.06 (7.6;10.9)	
	T/T	8.80 (7.7; 10.34)		9.10 (7.9;11.0)		8.55 (7.5; 10.0)	
rs4279640 T>C	T/T	9.00 (7.70; 10.8)	0.93	9.10 (7.7; 10.6)	0.66	9.00 (7.6; 10.8)	0.92
	T/C	9.10 (7.70; 10.9)		9.45 (8.0; 11.1)		9.00 (7.7; 10.8)	
	C/C	9.00 (7.9;10.5)		9.10 (7.9; 11.0)		9.00 (7.85; 10.4)	
rs3757971 T>C	T/T	9.00 (7.8; 10.6)	0.32	9.15 (7.7;10.6)	0.18	9.00 (7.9; 10.6)	0.61
	T/C	9.10 (7.7;11.0)		9.50 (7.9; 11.2)		9.00 (7.9;10.8)	
	C/C	8.80 (7.7; 10.5)		9.05 (7.9; 10.5)		8.60 (7.05;10.1)	
Fasting blood glucose							
rs7838717 C>T	C/C	12.10 (9.9; 15.0)	0.62	11.80 (9.2; 15.0)	0.11	12.20 (10.0; 15.0)	0.52
	C/T	12.35 (9.5; 15.4)		12.80 (9.7; 15.4)		12.00 (9.4; 15.4)	
	T/T	12.00 (9.59; 15.0)		12.60 (10.0; 15.4)		11.70 (9.3; 14.9)	
rs4279640 T>C	T/T	12.00 (9.6; 15.1)	0.87	12.60 (9.9; 15.2)	0.72	12.00 (9.4; 15.0)	0.55
	T/C	12.10 (9.6; 15.3)		12.60 (9.4; 15.2)		12.00 (9.6; 15.4)	
	C/C	12.20 (9.9; 15.0)		12.10 (9.5; 15.6)		12.25 (10.1; 15.0)	
rs3757971 T>C	T/T	12.10 (9.9; 15.0)	0.75	11.90 (9.2; 15.0)	0.12	12.20 (10.0; 15.1)	0.48
	T/C	12.25 (9.5; 15.4)		12.75 (9.7; 15.4)		12.00 (9.5; 15.4)	
	C/C	12.00 (9.5; 15.1)		12.50 (10.0; 15.5)		12.00 (9.3; 15.0)	
Glucose after breakfast							
rs7838717 C>T	C/C	6.69 (5.7; 7.8)	**0.027**	6.50 (5.5; 7.6)	0.054	6.70 (5.7; 7.8)	0.25
	C/T	6.50 (5.6; 7.6)		6.60 (5.6; 7.6)		6.50 (5.5; 7.6)	
	T/T	6.30 (5.5; 7.7)		5.90 (5.4; 7.3)		6.38 (5.5; 8.0)	
rs4279640 T>C	T/T	6.37 (5.5; 7.7)	0.22	6.30 (5.4; 7.6)	0.82	6.40 (5.6; 7.8)	0.26
	T/C	6.50 (5.6; 7.6)		6.43 (5.6; 7.4)		6.50 (5.6; 7.6)	
	C/C	6.70 (5.6; 7.9)		6.50 (5.5; 7.7)		6.87 (5.6; 7.9)	
rs3757971 T>C	T/T	6.70 (5.6; 7.8)	0.084	6.55 (5.3; 7.6)	0.14	6.70 (5.6; 7.8)	0.34
	T/C	6.50 (5.6; 7.6)		6.43 (5.6; 7.6)		6.50 (5.5; 7.6)	
	C/C	6.36 (5.6; 7.8)		6.00 (5.5; 7.6)		6.50 (5.7; 8.0)	
C- peptide							
rs7838717 C>T	C/C	2.53 (1.4; 3.4)	0.78	2.46 (1.2; 3.3)	0.67	2.76 (1.4; 3.7)	0.74
	C/T	2.24 (1.5; 3.2)		2.23 (1.6; 3.0)		2.28 (1.5; 3.5)	
	T/T	2.73 (1.3; 3.8)		2.68 (1.2; 4.4)		2.73 (1.4; 3.5)	
	T/T	2.49 (1.2; 3.8)	0.68	2.41 (1.2; 3.8)	0.87	2.53 (1.4; 3.7)	0.60

rs4279640 T>C	T/C	2.28 (1.5; 3.2)		2.19 (1.5; 3.1)		2.29 (1.5; 3.3)	
	C/C	2.46 (1.5; 3.4)		2.23 (1.4; 3.3)		2.53 (2.1; 4.3)	
rs3757971 T>C	T/T	2.46 (1.4; 3.4)		2.46 (1.4; 3.4)		2.50 (1.5; 3.7)	
	T/C	2.28 (1.5; 3.1)	0.85	2.15 (1.5; 3.0)	0.88	2.31 (1.5; 3.3)	0.94
	C/C	2.57 (1.2; 3.9)		2.40 (1.3; 3.8)		2.73 (1.0; 4.0)	
				ROS			
rs7838717 C>T	C/C	3.64 (2.7; 5.1)		3.18 (2.5; 4.8)		3.80 (2.9; 5.1)	
	C/T	3.81 (2.7; 5.0)	0.13	3.63 (2.4; 4.8)	0.56	3.87 (2.8; 5.0)	0.12
	T/T	3.41 (2.3; 4.7)		2.98 (2.3; 4.8)		3.44 (2.3; 4.7)	
rs4279640 T>C	T/T	3.77 (2.6; 4.9)	0.74	3.92 (2.7; 5.1)		3.73 (2.6; 4.9)	
	T/C	3.62 (2.6; 5.0)		3.27 (2.4; 4.4)	0.27	3.71 (2.7; 5.1)	0.34
	C/C	3.84 (2.7; 5.0)		3.10 (2.5; 4.8)		4.03 (3.0; 5.2)	
rs3757971 T>C	T/T	3.80 (2.7; 5.1)		3.54 (2.5; 5.2)		3.89 (2.8; 5.1)	
	T/C	3.70 (2.6; 4.9)	0.33	3.45 (2.3; 4.5)	0.96	3.79 (2.8; 5.0)	0.21
	C/C	3.45 (2.3; 4.8)		3.33 (2.3; 4.7)		3.45 (2.4; 5.0)	
				GSH			
rs7838717 C>T	C/C	0.85 (0.4; 1.6)		0.70 (0.4; 1.7)		0.87 (0.4; 1.5)	
	C/T	0.84 (0.5; 1.4)	0.99	0.92 (0.4; 1.4)	0.40	0.79 (0.5; 1.3)	0.71
	T/T	0.76 (0.5; 2.2)		0.62 (0.2; 0.9)		1.01 (0.5; 2.6)	
rs4279640 T>C	T/T	0.81 (0.5; 1.3)		0.92 (0.5; 1.1)		0.73 (0.5; 1.4)	0.58
	T/C	0.82 (0.5; 1.4)	0.94	0.87 (0.3; 1.4)	0.72	0.80 (0.5; 1.4)	
	C/C	0.89 (0.5; 1.8)		0.70 (0.5; 1.7)		0.91 (0.5; 1.8)	
rs3757971 T>C	T/T	0.91 (0.5; 1.6)		0.70 (0.4; 1.7)		0.92 (0.5; 1.5)	
	T/C	0.71 (0.4; 1.4)	0.46	0.95 (0.4; 1.4)	0.66	0.65 (0.4; 1.2)	0.064
	C/C	0.90 (0.7; 1.5)		0.74 (0.5; 0.9)		1.02 (0.7; 2.4)	
				GSSG			
rs7838717 C>T	C/C	4.16 (1.8; 5.5)		5.47 (2.3; 6.4)	0.51	3.01 (2.2; 3.0)	
	C/T	2.62 (1.9; 3.0)	0.13	1.87 (1.87; 1.87)		3.95 (1.2; 5.5)	0.23
	T/T	4.29 (2.0; 5.8)		-		4.29 (0.1; 5.8)	
rs4279640 T>C	T/T	3.48 (1.4; 5.1)		-		2.63 (1.8; 5.2)	0.54
	T/C	2.26 (1.8; 4.1)	0.14	2.08 (1.9; 2.3)	0.13	3.48 (1.4; 5.1)	
	C/C	5.47 (4.4; 6.4)		5.92 (5.5; 6.4)		4.38 (1.0; 6.5)	
rs3757971 T>C	T/T	4.38 (2.3; 5.5)		5.47 (2.3; 6.4)		2.62 (1.8; 3.0)	
	T/C	2.24 (1.8; 3.0)	0.06	1.87 (1.87; 1.87)	0.51	4.16 (1.2; 5.5)	0.11
	C/C	4.29 (2.1; 5.8)		-		4.29 (2.1; 5.8)	
				GSSG-CellB			
rs7838717 C>T	C/C	1.29 (0.5; 3.7)		1.64 (0.5; 3.7)		1.05 (0.4; 3.8)	
	C/T	2.00 (0.6; 3.9)	0.30	2.00 (0.9; 4.0)	0.91	2.00 (0.6; 3.9)	0.09
	T/T	1.35 (0.8; 3.5)		2.69 (1.2; 3.9)		1.19 (0.5; 1.9)	

rs4279640 T>C	T/T	1.95 (0.8; 3.7)	0.33	2.63 (1.2; 3.9)	0.55	1.93 (0.5; 3.5)	0.59
	T/C	1.36 (0.6; 3.7)		1.47 (0.6; 3.6)		1.35 (0.5; 3.7)	
	C/C	2.23 (0.5; 4.0)		2.27 (0.6; 4.3)		2.01 (0.4; 4.0)	
rs3757971 T>C	T/T	1.29 (0.5; 3.8)	0.37	1.88 (0.6; 3.7)	0.99	0.96 (0.4; 3.9)	0.12
	T/C	1.97 (0.6; 3.9)		1.55 (0.7; 3.9)		2.00 (0.6; 3.9)	
	C/C	1.31 (0.6; 3.4)		2.05 (1.2; 4.2)		1.19 (0.3; 2.2)	
Cholesterol							
rs7838717 C>T	C/C	4.98 (4.2; 6.0)	0.11	4.76 (4.0; 5.7)	0.18	5.10 (4.3; 6.1)	0.39
	C/T	5.18 (4.3; 6.2)		5.05 (4.1; 6.0)		5.31 (4.4; 6.2)	
	T/T	5.13 (4.3; 6.2)		4.87 (4.0; 5.8)		5.21 (4.4; 6.4)	
rs4279640 T>C	T/T	5.20 (4.2; 6.2)	0.35	5.10 (4.0; 6.0)	0.70	5.25 (4.3; 6.2)	0.75
	T/C	5.09 (4.3; 6.1)		4.90 (4.1; 5.8)		5.18 (4.5; 6.2)	
	C/C	5.00 (4.1; 6.1)		4.86 (4.0; 5.8)		5.17 (4.2; 6.3)	
rs3757971 T>C	T/T	5.00 (4.2; 6.0)	0.19	4.80 (4.0; 5.7)	0.30	5.15 (4.3; 6.2)	0.37
	T/C	5.13 (4.3; 6.1)		5.07 (4.1; 6.0)		5.20 (4.4; 6.2)	
	C/C	5.21 (4.2; 6.4)		4.87 (4.0; 5.8)		5.40 (4.4; 6.5)	
Low density lipoproteins							
rs7838717 C>T	C/C	2.90 (2.4; 3.9)	0.33	2.80 (2.4; 3.5)	0.64	3.11 (2.5; 4.3)	0.14
	C/T	3.12 (2.4; 4.1)		2.68 (2.0; 3.4)		3.50 (2.7; 4.5)	
	T/T	3.11 (2.3; 4.0)		2.65 (2.0; 4.0)		3.19 (2.5; 4.4)	
rs4279640 T>C	T/T	2.90 (2.3; 4.1)	0.49	2.55 (1.8; 3.8)	0.54	3.32 (2.6; 4.6)	0.15
	T/C	3.10 (2.4; 4.1)		2.74 (2.2; 3.4)		3.44 (2.7; 4.4)	
	C/C	2.90 (2.4; 3.8)		2.80 (2.6; 3.7)		3.10 (2.3; 4.2)	
rs3757971 T>C	T/T	2.98 (2.4; 3.9)	0.56	2.80 (2.3; 3.6)	0.37	3.13 (2.5; 4.3)	**0.033**
	T/C	3.10 (2.4; 4.1)		2.68 (2.0; 3.4)		3.50 (2.7; 4.5)	
	C/C	2.90 (2.3; 4.0)		2.70 (2.0; 4.0)		3.16 (2.5; 4.4)	
High density lipoproteins							
rs7838717 C>T	C/C	0.85 (0.7; 1.1)	0.82	0.85 (0.7; 1.0)	0.70	0.84 (0.7; 1.0)	0.80
	C/T	0.85 (0.7; 1.1)		0.89 (0.7; 1.1)		0.85 (0.7; 1.1)	
	T/T	0.84 (0.8; 1.0)		0.85 (0.7; 1.0)		0.84 (0.8; 1.0)	
rs4279640 T>C	T/T	0.84 (0.7; 1.1)	0.46	0.88 (0.7; 1.1)	0.95	0.87 (0.8; 1.1)	0.23
	T/C	0.85 (0.7; 1.1)		0.84 (0.7; 1.1)		0.82 (0.7; 1.0)	
	C/C	0.86 (0.8; 1.0)		0.88 (0.8; 1.0)		0.85 (0.8; 1.0)	
rs3757971 T>C	T/T	0.85 (0.7; 1.1)	0.93	0.85 (0.7; 1.0)	0.68	0.84 (0.7; 1.0)	0.60
	T/C	0.84 (0.7; 1.1)		0.89 (0.7; 1.1)		0.85 (0.8; 1.1)	
	C/C	0.84 (0.8; 1.0)		0.86 (0.7; 1.0)		0.84 (0.8; 1.1)	
Triglycerides							
rs7838717	C/C	2.14 (1.5; 3.0)	0.54	2.10 (1.4; 2.8)	0.44	2.16 (1.5; 3.0)	0.38
	C/T	2.21 (1.6; 3.0)		2.16 (1.4; 2.9)		2.26 (1.7; 3.0)	

C>T	T/T	2.20 (1.6; 3.1)		2.38 (1.6; 3.2)		2.16 (1.6; 2.9)	
rs4279640 T>C	T/T	2.20 (1.6; 3.1)	0.35	2.20 (1.5; 3.1)	0.43	2.20 (1.7; 3.0)	**0.039**
	T/C	2.21 (1.6; 3.1)		2.03 (1.4; 2.9)		2.34 (1.7; 3.1)	
	C/C	2.12 (1.5; 2.8)		2.22 (1.6; 3.0)		2.05 (1.5; 2.7)	
rs3757971 T>C	T/T	2.14 (1.5; 3.0)	0.53	2.11 (1.4; 2.8)	0.57	2.15 (1.5; 3.1)	0.60
	T/C	2.20 (1.6; 3.0)		2.16 (1.4; 3.0)		2.25 (1.7; 3.0)	
	C/C	2.23 (1.6; 3.1)		2.22 (1.6; 3.1)		2.24 (1.6; 3.0)	
Urea							
rs7838717 C>T	C/C	6.00 (4.5; 8.0)	0.16	5.55 (4.2; 7.5)	0.58	6.07 (4.8; 8.1)	0.19
	C/T	6.45 (5.0; 8.9)		6.40 (5.0; 8.9)		6.50 (5.0; 8.8)	
	T/T	6.90 (5.5; 8.2)		6.40 (5.5; 8.0)		6.90 (5.3; 9.2)	
rs4279640 T>C	T/T	6.50 (5.1; 8.7)	0.48	6.30 (5.0; 8.1)	0.27	6.70 (5.2; 8.9)	0.49
	T/C	6.30 (5.0; 8.6)		6.50 (5.0; 8.3)		6.30 (5.0; 8.6)	
	C/C	5.85 (4.5; 8.0)		5.35 (4.1; 8.0)		6.40 (4.7; 8.0)	
rs3757971 T>C	T/T	5.90 (4.5; 8.0)	0.15	5.25 (4.1; 7.1)	0.08	6.00 (4.8; 8.2)	0.25
	T/C	6.50 (5.0; 8.8)		6.75 (5.3; 9.0)		6.40 (4.8; 8.8)	
	C/C	6.75 (5.3; 8.2)		6.30 (5.2; 7.9)		6.85 (5.3; 8.7)	
Uric acid							
rs7838717 C>T	C/C	332.14 (253.3; 406.0)	0.65	330.86 (251.9; 403.0)	0.64	333.41 (253.7; 410.3)	0.55
	C/T	327.08 (259.7; 399.9)		335.06 (282.0; 376.7)		322.98 (251.2; 407.0)	
	T/T	311.17 (263.4; 390.5)		367.09 (294.2; 414.3)		303.61 (261.7; 377.7)	
rs4279640 T>C	T/T	323.22 (251.7; 387.9)	0.43	325.83 (268.2; 400.8)	0.88	322.35 (251.2; 378.1)	0.56
	T/C	326.93 (253.7; 396.3)		335.99 (280.5; 383.6)		318.40 (248.3; 402.0)	
	C/C	340.00 (270.1; 411.9)		344.63 (263.1; 402.5)		339.94 (274.8; 420.3)	
rs3757971 T>C	T/T	339.00 (259.6; 410.2)	0.50	342.93 (267.6; 402.1)	0.75	338.35 (256.1; 412.6)	0.35
	T/C	322.98 (251.1; 385.7)		334.66 (268.2; 376.7)		317.43 (246.8; 386.4)	
	C/C	324.65 (270.9; 401.3)		361.59 (294.2; 414.3)		311.31 (268.8; 387.9)	
Creatinin							
rs7838717 C>T	C/C	96.00 (85.0; 112.0)	0.50	99.50 (88.0; 115.)	0.56	92.00 (84.0; 110.0)	0.11
	C/T	95.00 (82.0; 111.0)		98.00 (86.9; 115.0)		93.00 (80.0; 108.0)	
	T/T	92.00 (81.0; 107.0)		101.00 (90.0; 114.0)		89.00 (77.5; 103.0)	
rs4279640 T>C	T/T	94.00 (82.0; 108.0)	0.08	99.00 (89.0; 112.0)	0.90	91.50 (80.0; 107.0)	0.22
	T/C	94.00 (82.0; 111.0)		99.00 (86.9; 118.0)		91.00 (79.0; 107.0)	
	C/C	98.00 (85.0; 112.0)		100.00 (90.0; 113.0)		97.00 (84.0; 110.0)	
rs3757971 T>C	T/T	96.00 (85.0; 111.0)	0.72	100.00 (89.0; 114.0)	0.30	92.00 (84.0; 109.0)	0.56
	T/C	94.00 (81.0; 111.0)		97.00 (86.9; 117.0)		93.00 (78.0; 108.0)	
	C/C	95.00 (82.0; 109.0)		101.00 (90.0; 114.0)		90.50 (80.0; 105.0)	

Glomerular filtration rate							
rs7838717 C>T	C/C	61.00 (51.0; 73.9)	0.41	68.50 (59.0; 80.0)	0.67	57.39 (47.0; 67.0)	0.45
	C/T	62.00 (50.0; 76.2)		71.00 (60.2; 83.0)		58.00 (48.0; 71.0)	
	T/T	62.00 (50.5; 74.0)		71.76 (60.0; 81.3)		60.25 (49.0; 70.4)	
rs4279640 T>C	T/T	65.00 (53.9; 75.0)	**0.035**	72.97 (61.8; 82.0)	0.22	60.39 (50.0; 70.0)	**0.021**
	T/C	60.95 (50.0; 75.0)		67.00 (55.0; 80.0)		58.00 (48.6; 72.0)	
	C/C	60.46 (49.0; 75.0)		71.00 (60.0; 83.0)		54.80 (45.0; 65.0)	
rs3757971 T>C	T/T	60.98 (50.0; 73.9)	0.17	69.00 (59.0; 80.0)	0.86	57.45 (46.7; 66.0)	0.37
	T/C	62.00 (50.8; 76.0)		70.64 (60.0; 83.5)		57.75 (48.5; 71.5)	
	C/C	63.50 (51.0; 75.0)		71.26 (60.0; 81.6)		60.39 (49.0; 71.0)	
Daily Protein							
rs7838717 C>T	C/C	0.03 (0.03; 0.03)	0.73	0.03 (0.03; 0.03)	0.82	0.03 (0.03; 0.03)	0.81
	C/T	0.03 (0.03; 0.03)		0.03 (0.03; 0.06)		0.03 (0.03; 0.03)	
	T/T	0.03 (0.03; 0.06)		0.03 (0.03; 0.06)		0.03 (0.03; 0.05)	
rs4279640 T>C	T/T	0.03 (0.03; 0.033)	0.86	0.03 (0.03; 0.03)	0.96	0.03 (0.03; 0.06)	0.67
	T/C	0.03 (0.03; 0.03)		0.03 (0.03; 0.06)		0.03 (0.03; 0.03)	
	C/C	0.03 (0.03; 0.03)		0.03 (0.03; 0.06)		0.03 (0.03; 0.03)	
rs3757971 T>C	T/T	0.03 (0.03; 0.03)	0.81	0.03 (0.03; 0.03)	0.89	0.03 (0.03; 0.03)	0.87
	T/C	0.03 (0.03; 0.032)		0.03 (0.03; 0.03)		0.03 (0.03; 0.033)	
	C/C	0.03 (0.03; 0.05)		0.03 (0.03; 0.06)		0.03 (0.03; 0.033)	
Insulin							
rs7838717 C>T	C/C	-	0.51	-	0.06	-	0.69
	C/T	19.40 (10.3; 35.4)		19.40 (19.4; 19.4)		22.85 (10.3; 35.4)	
	T/T	21.00 (21.0; 21.0)		-		21.00 (21.0; 21.0)	
rs4279640 T>C	T/T	20.20 (19.4; 21.0)	1.00	19.40 (19.4; 19.4)	1.00	21.00 (21.0; 21.0)	0.68
	T/C	22.85 (10.3; 35.4)		-		22.85 (10.3; 35.4)	
	C/C	-		-		-	
rs3757971 T>C	T/T	-	0.51	-	1.00	-	0.69
	T/C	19.40 (10.3; 35.4)		19.40 (19.4; 19.4)		22.85 (10.3; 35.4)	
	C/C	21.00 (21.0; 21.0)		-		21.00 (21.0; 21.0)	

[1]Data are presented as median, first and third quartiles

[2] p-values for the Kruskal–Wallis one-way analysis of variance.

Bold indicates statistically significant p- and Q-values.
